# ATPase Inhibitory Factor 1 Is Critical for Regulating Sevoflurane-Induced Microglial Inflammatory Responses and Caspase-3 Activation

**DOI:** 10.3389/fncel.2021.770666

**Published:** 2021-12-15

**Authors:** Yaru Xu, Ge Gao, Xiaoru Sun, Qidong Liu, Cheng Li

**Affiliations:** ^1^Department of Anesthesiology and Perioperative Medicine, Shanghai Fourth People’s Hospital, School of Medicine, Tongji University, Shanghai, China; ^2^Translational Research Institute of Brain and Brain-Like Intelligence, Shanghai Fourth People’s Hospital, School of Medicine, Tongji University, Shanghai, China; ^3^Clinical Research Center for Anesthesiology and Perioperative Medicine, Tongji University, Shanghai, China; ^4^Department of Anesthesiology, Shanghai Tenth People’s Hospital, School of Medicine, Tongji University, Shanghai, China; ^5^Center for Translational Neurodegeneration and Regenerative Therapy, Shanghai Tenth People’s Hospital, School of Medicine, Tongji University, Shanghai, China; ^6^Anesthesia and Brain Research Institute, Shanghai Tenth People’s Hospital, School of Medicine, Tongji University, Shanghai, China; ^7^Key Laboratory of Spine and Spinal Cord Injury Repair and Regeneration of Ministry of Education, Orthopedic Department of Tongji Hospital, School of Medicine, Tongji University, Shanghai, China

**Keywords:** sevoflurane, postoperative delirium (POD), microglia, ATPIF1, ATP – adenosine triphosphate

## Abstract

Postoperative delirium (POD) is one of the most important complications after surgery with general anesthesia, for which the neurotoxicity of general anesthetics is a high-risk factor. However, the mechanism remains largely unknown, which also hinders the effective treatment of POD. Here, we confirmed that a clinical concentration of the general anesthetic sevoflurane increased the expression of inflammatory factors and activated the caspase-3 by upregulating ATPase inhibitory factor 1 (ATPIF1) expression in microglia. Upregulation of ATPIF1 decreased the synthesis of ATP which is an important signaling molecule secreted by microglia. Extracellular supplementation with ATP attenuated the microglial inflammatory response and caspase-3 activation caused by sevoflurane or overexpression of ATPIF1. Additionally, the microglial inflammatory response further upregulated ATPIF1 expression, resulting in a positive feedback loop. Animal experiments further indicated that intraperitoneal injection of ATP significantly alleviated sevoflurane anesthesia-induced POD-related anxiety behavior and memory damage in mice. This study reveals that ATPIF1, an important protein regulating ATP synthesis, mediates sevoflurane-induced neurotoxicity in microglia. ATP supplementation may be a potential clinical treatment to alleviate sevoflurane-induced POD.

## Introduction

Postoperative delirium (POD) is a frequent manifestation of acute cerebral dysfunction in older adults after surgery under general anesthesia that usually peaks between 1 and 3 days after the operation (Al Tmimi et al., 2015; Oh and Park, 2019). Although evidences indicated that POD is associated with short-term complications, long-term sequelae may also be induced after the POD (Wacker et al., 2006; Whitlock et al., 2011). For instance, Inouye et al. concluded that there is a significant acceleration of cognitive deficit following an episode of POD, which suggests that there is a strong correlation between the occurrence of POD and long-term risk of dementia (Fong et al., 2009). Additionally, it has been reported that POD can arise in the same individuals with overlapping risk factors, leading to a common underlying neuropathogenesis of postoperative cognitive dysfunction (POCD) (Fong et al., 2009; Schmitt et al., 2012). Given the harm of POD to patients, it is important to determine the mechanism of POD to inform further treatment.

The available evidence demonstrates that central nervous system dysfunction caused by anesthesia during surgery that further leads to neurocognitive disorders is a main factor related to POD (Evered and Silbert, 2018). The original validation study showed that individuals who underwent general anesthesia had a 40% higher risk of incident dementia than those who were not hospitalized for surgery (Ehlenbach et al., 2010). More specifically, several lines of evidence have established that cognitive dysfunction can be observed in rats and mice in anesthesia-only groups (Bianchi et al., 2008; Kawano et al., 2018). Clinically, the incidence of POD is higher in geriatric surgical patients under inhalational anesthesia than in those under intravenous anesthesia, whereas elderly patients undergoing major surgery under sevoflurane inhalation anesthesia develop amnestic mild cognitive impairment at a faster rate (Qiao et al., 2015). The issue of general anesthetic-induced neurotoxicity has continued to garner attention in recent years (Vlisides and Xie, 2012). Nevertheless, the underlying mechanisms by which general anesthesia induces POD remain largely unclear.

It has been illustrated that anesthetic lesions dominate the primary inflammatory response, which gives rise to neuroinflammation and increases the risk of developing POD (Clarke et al., 2008; Pac-Soo et al., 2011). Given the importance of neuroinflammation in POD, targeting inflammatory processes should be considered as an approach for the prevention and treatment of POD. Neuroinflammation modulated by microglia has been implicated in delirium, and anesthetics may also be involved in inflammatory responses in microglia, influencing the development of delirium (Riker et al., 2009; Peng et al., 2013; Djaiani et al., 2016). Importantly, adenosine triphosphate (ATP) serves as an apparently ubiquitous “gliotransmitter” that can be released by microglia, regulating the functions of microglia as well as neurons, especially in pathophysiological conditions (Imura et al., 2013). A previous study has indicated that isoflurane decreases the ATP concentration in the mouse brain (Wang et al., 2015). Sevoflurane can also decrease ATP production in neurons (Zhu et al., 2021). However, the mechanism by which sevoflurane regulates ATP production remains largely unclear.

Inhalation anesthesia has been reported to elicit mitochondrial dysfunction, leading to neuronal apoptosis (Swerdlow et al., 2000; Jha and Morrison, 2018; Chen et al., 2020). As a mitochondria-localized protein, ATPase inhibitory factor 1 (ATPIF1) contributes to the maintenance of homeostasis and the normal functions of mitochondria (Campanella et al., 2008; Faccenda et al., 2017). Under physiological conditions, ATPIF1 binds to the β subunits of the F1 domain of ATP synthase, preventing the hydrolytic activity of F1 and hence maintaining the intracellular ATP level (Bonora et al., 2015). Nevertheless, overexpression of ATPIF1 significantly reduces mitochondrial membrane potential, leading to mitochondrial dysfunction and decreased ATP synthesis followed by activation of the mitochondrial apoptotic pathway (Chen et al., 2014). Although it is known that maintaining normal ATPIF1 expression is essential for mitochondrial and cell survival, the understanding of whether general anesthesia affects ATPIF1 to regulate downstream signaling pathways leading to POD pathogenesis is rather limited.

Here, we confirmed that a clinical concentration of general anesthetic sevoflurane increased the expression of inflammatory factors in microglia and activated the caspase-3 by upregulating ATPIF1 expression in microglia. Upregulation of ATPIF1 in microglia decreased the synthesis of ATP, which is also an important secreted signaling molecule. Sevoflurane treatment or overexpression of ATPIF1 induced a microglial inflammatory response and caspase-3 activation, which was able to be attenuated by extracellular supplementation of ATP. In addition, the microglial inflammatory response further upregulated ATPIF1 expression, resulting in a positive feedback loop. Animal experiments further indicated that intraperitoneal injection of ATP significantly alleviated the anxiety and memory damage induced by sevoflurane anesthesia in a POD mouse model, which provides a potential convenient strategy for the future clinical treatment of POD.

## Materials and Methods

### Mice and Postoperative Delirium Model Establishment

The animal-related protocol was performed according to the Standing Committee on Animals at the Shanghai Tenth People’s Hospital, and C57BL/6 mice were maintained according to the guidelines in the laboratory animal center at Tongji University.

Postoperative delirium may occur more in female patients (Vaurio et al., 2006; Tagarakis et al., 2007). So, we employed the female mice for study. Previous study also indicated that surgery could induce the inflammatory reaction to potentiate the neurotoxicity (Ren et al., 2015; Peng et al., 2016; Yang et al., 2017), which will interfere us to judge whether sevoflurane causes POD related behavior and inflammatory response of microglia in brain. So, we established the mice model that only received clinical concentration 3% sevoflurane (40% oxygen and 57% nitrogen) for 6 h anesthesia. The control mice received 40% oxygen and 60% nitrogen. The temperature was controlled to maintain at 30°C during the anesthesia. For immunofluorescence staining of Iba1 in mice brain, three mice were employed in each group. For behavior test, eight mice were employed in each group.

### Isolation of Primary Microglia

Mouse primary microglia were isolated from postnatal day 1 (P1) C57BL/6 mouse brains. The mice were sacrificed to dissect out the brains. The brain of each mouse was digested in 0.25% trypsin solution with 0.05% DNase I at 37°C for half an hour and then incubated with 10% fetal bovine serum (FBS) (Lonsera, S711-001S, Uruguay). The tissue sediment was centrifuged at 1000 rpm for 5 min at 4°C and washed twice with HBSS. After trituration, the cells were plated and cultured in DMEM with 10 ng/mL GM-CSF supplemented with 10% FBS, 50 U penicillin and 50 mg/mL streptomycin at 37°C. The culture dishes or plates were coated with 100 μg/mL poly-D-lysine (Sigma) and 5 μg/mL fibronectin (Sigma). The culture medium was replaced every 3 days. The cells were subjected to three passages for purification purposes. Microglial purity was confirmed by immunostaining with a primary antibody against Iba1 (Synaptic Systems, 234003).

### Immunofluorescence Staining

Mice were killed after 6 h of anesthesia. The treatment and control mice were both deeply anesthetized and transcardially perfused with 30 ml of 0.01 M PBS (pH 7.4) followed by 100 ml of 4% (w/v) paraformaldehyde (PFA). The brains were isolated and postfixed with 4% PFA for 4 h at 4 °C. After cryoprotection with 30% (w/v) sucrose in 0.1 M phosphate buffer at 4 °C overnight, the brains were cut into 30 μm thick frontal sections using a freezing microtome. The sections were incubated overnight at 4 °C with a mouse Iba1 antibody (1:200 dilution, Synaptic Systems, 234003). The sections were washed and incubated for 2 h with anti-rabbit IgG (Alexa Fluor^®^ 488 Conjugate) (Cell Signaling Technology, #4412) at room temperature to detect microglia in the hippocampus. Images were obtained with a confocal microscope (Olympus, FV3000, Japan).

### Knockdown of Mouse ATPase Inhibitory Factor 1 in BV2 Cell

The mouse microglial cell line BV2 was infected with a pLKD-CMV-puro-U6-ATPIF1 shRNA lentivirus (Obio, China) to knock down ATPIF1 expression. The sequences were as follows: shRNA-1: 5′-CCGGCCATAAGAAGAAGATCCAATTCAAGAGATTGGAT CTTCTTCTTATGGTTTTTTG-3′; shRNA-2: 5′-CCGGGC GTCTGCAGAAGCAAATTCTCAAGAGAAATTTGCTTCTGC AGACGCTTTTTTG-3′; and scramble control shRNA: 5′-CCGGTTCTCCGAACGTGTCACGTTTCAAGAGAACGTGA CACGTTCGGAGAATTTTTTG-3′. The stable cell lines were selected with puromycin (puro, Sigma, United States) for a week.

### Overexpression of ATPase Inhibitory Factor 1 in BV2 Cell

The mouse microglial cell line BV2 was infected with a pGMLV-CMV-ATPIF1-puro lentivirus (Genomeditech, China) to overexpress ATPIF1. The control cell was infected with the empty vector virus (Genomeditech). Stable cell line overexpressing ATPIF1 or control was selected with puromycin (puro) for a week.

### Cell Treatment

The different groups of treatment of cells: 3% sevoflurane with 5% CO_2_, dexamethasone sodium phosphate injection (DXM) (1:1000 dilution, Hasen-modern), methylprednisolone sodium succinate for injection (MPSS) (1:100 dilution, Pharmacia & Upjohn N.V./S.A.), lipopolysaccharide, (LPS, 100 ng/ml, Sigma), TNF-α (10 ng/ml, Sigma), or ATP (50 μg/ml, Sigma).

### Western Blot

Different treatment groups of cells were lysed by using protein lysis buffer (M-PER Mammalian Protein Extraction Reagent, Thermo Fisher Scientific, United States) with protease inhibitors (Bimake, China). Before protein electrophoresis, we performed protein quantification by using BCA protein concentration determination kit (Beyotime, China). Equal amount of each sample (10 μg of protein) was used for detection. The whole proteins were transferred onto PVDF membranes (Bio-Rad, United States). The primary antibodies included a β-Actin antibody (1:1000 dilution, #4967, Cell Signaling Technology, United States), a Caspase-3 antibody (1:1000 dilution, #9662, Cell Signaling Technology), a Cleaved caspase-3 (C-Caspase-3) antibody (1:1000 dilution, #9664, Cell Signaling Technology), an ATPIF1 antibody (1:1000 dilution, ab110277, Abcam, United States), a COX-2 antibody (1:1000 dilution, ab15191, Abcam), and a TNF-α antibody (1:1000 dilution, ab1793, Abcam). The secondary antibodies were a rabbit IgG HRP-linked antibody (1:3000 dilution, 7074S, Cell Signaling Technology) and a mouse IgG HRP-linked antibody (1:3000 dilution, 7076S, Cell Signaling Technology). A ChemiDoc XRS + system (Bio-Rad) was used for detection of protein expression by enhanced chemiluminescence (Clarity Western ECL Substrate, Bio-Rad). β-Actin was used to normalize the target protein levels (e.g., ratio of Caspase-3 to β-Actin amount was used to determine the relative expression level of Caspase-3 in different group). β-Actin also controls for loading differences in the total protein amount.

### Detection of the Level of Intracellular Adenosine Triphosphate

The levels of intracellular ATP in the microglial cells in each group were detected with an ATP assay kit (Abcam, ab83355). The microglia in each group were lysed with assay buffer, and then the sample supernatant was isolated by centrifugation (12,000 × *g*) at 4°C for 5 min. Then, 50 μl of ATP reaction mix and 50 μl of cell supernatant sample were added to a 96-well plate, and the plate was incubated at room temperature for 30 min while protected from light. The output was measured on a microplate reader to determine the OD at 570 nm.

### Open Field Test

The open field test used to test anxious behavior in mice was performed as previously described (Han et al., 2012). Each individual mouse in the different groups was placed near the wall of the open-field arena, and the movements of the mouse in 10 min were recorded with ANY-maze software (Stoelting, United States).

### Novel Object Recognition Test

The novel object recognition test used for testing learning and memory was performed as previously described (Han et al., 2012). The arena was used once for each mouse. During the habituation session, the test mouse was placed in the arena to explore for 10 min. Following habituation, two objects of similar size but different shape and color were placed in the opposite corners of the arena. Then, the test mouse was placed in the center and allowed to explore for 10 min. Twenty-four hours later, one object was replaced, and the same test mouse was allowed to explore. The movement of the mouse was recorded and analyzed with ANY-maze (Stoelting).

### Y-Maze Test

The Y-maze test used for testing spatial learning and memory was performed as previously described (Paretkar and Dimitrov, 2018; Li et al., 2019). Each mouse was placed at the end of one arm and allowed to explore the arms freely for 5 min. The percentage of “correct” spontaneous alternations was calculated.

### Statistical Analyses

The data were analyzed using Student’s *t*-test for comparisons of the control and treatment groups. One-way ANOVA was used to determine the significance between different treatments. The data are reported as the means ± SD. The results were considered significantly different when *P* < 0.05. Statistical analysis was conducted by using GraphPad Prism software (version 8.0; GraphPad Software, La Jolla, CA, United States).

## Results

### Sevoflurane Induces Inflammation and Caspase-3 Activation and Decreases the Adenosine Triphosphate Concentration in Microglia

**FIGURE 1:**
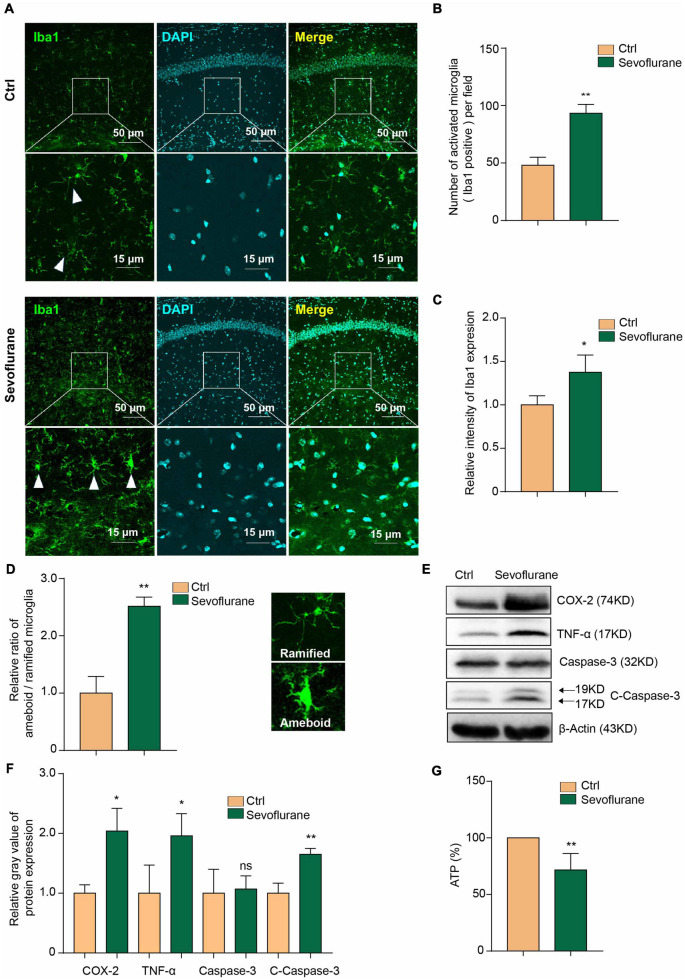
Sevoflurane induces inflammation and decreases the ATP concentration in microglia. **(A)** Microglial cells were activated in the mouse hippocampus after 3% sevoflurane treatment, as detected by Iba1 immunofluorescence staining. The scale bar indicates 50 (upper row) and 15 μm (lower row), respectively. **(B)** Quantification of Iba1 staining positive (Iba1 +) cell numbers and **(C)** intensity in hippocampal tissue from control- and sevoflurane-treated mice. **(D)** We morphologically classified the activated (amoeboid) or surveying (ramified) cells of Iba1 positive (Iba1 +) microglia to further perform the statistics of the ratio between amoeboid/ramified. **(E)** Western blot analysis indicated that 3% sevoflurane treatment for 6 h induced upregulation of the inflammatory factors COX-2 and TNF-α and activation of caspase-3 in mouse primary microglia. **(F)** Statistics of the relative gray value indicating the protein expression in panel **(E)**. **(G)** The concentration of ATP was decreased in sevoflurane-treated microglia. Ctrl means control. β-Actin served as a loading control. Student’s *t*-test was used to determine differences in panels **(B–D,F,G)**. The data shown are the means ± SD, *n* = 3 in panels **(B–D,F)**. *n* = 4 in panel **(G)**. * or ** means *p* < 0.05 or 0.01. ns means no significant difference compared with the control group.

Fourteen months old aged mice were treated with 3% sevoflurane for 6 h to establish the POD mouse model. We confirmed the similar phenomenon of previous study showing that sevoflurane induces microglial activation (Wang et al., 2021). We performed the immunofluorescence staining of Iba1 (Figure 1A), a recognized maker for microglia, to detect the microglia activation and found there’s significant increased Iba1 + cell numbers (Figure 1B) and microglial intensity (Figure 1C) in sevoflurane mice hippocampus. We also morphologically classified the activated (amoeboid) or surveying (ramified, not activated) cells (Thored et al., 2009) in Iba1 positive (Iba1 +) microglia to further perform the statistics of the ratio between amoeboid/ramified. In the sevoflurane treating mice, there’s higher ratio of amoeboid/ramified than control group (Figure 1D). We treated mouse primary microglia with 3% sevoflurane for 6 h and detected that the inflammatory mediators COX-2 and TNF-α were upregulated (Figures 1E,F). Additionally, we found that sevoflurane induced the caspase-3 activation (Figures 1E,F).

Previous studies have indicated that isoflurane decreases ATP levels in the mouse brain (Wang et al., 2015). Sevoflurane can also decrease ATP production in neurons (Zhu et al., 2021). Here, we further found that the concentration of ATP was decreased in microglia by sevoflurane treatment (Figure 1G). This result confirms that sevoflurane induces microglial inflammatory activation and caspase-3 activation and decreases the ATP concentration.

### ATPase Inhibitory Factor 1 Mediates Sevoflurane-Induced Inflammation and Caspase-3 Activation

**FIGURE 2:**
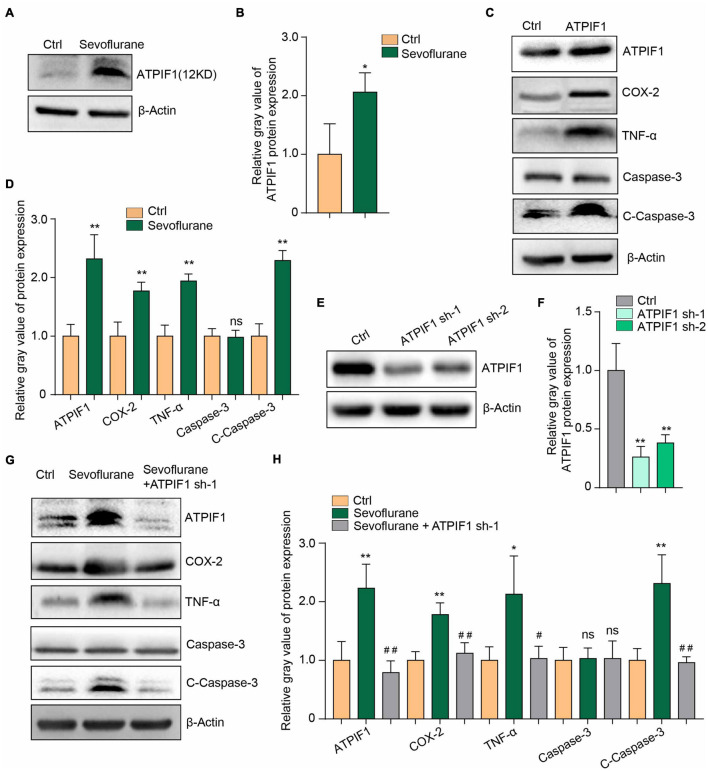
ATPIF1 mediates sevoflurane-induced inflammation and caspase-3 activation in microglia. **(A)** Representative western blot image indicating that ATPIF1 expression was upregulated by sevoflurane treatment in mouse primary microglia. **(B)** Statistics of the relative gray value indicating the ATPIF1 protein expression in panel **(A)**. **(C)** Overexpression of ATPIF1 induced inflammation and caspase-3 activation in BV2 cells. **(D)** Statistics of the relative gray value indicating the protein expression in panel **(C)**. **(E)** Downregulation of ATPIF1 expression by shRNA in BV2 cells. **(F)** Statistics of the relative gray value indicating the ATPIF1 protein expression in panel **(E)**. **(G)** Downregulation of ATPIF1 attenuated the ATPIF1 upregulation caused by treatment with sevoflurane and attenuated the expression of the inflammatory factors COX-2 and TNF-α and the activation of caspase-3 to levels similar to those in the control group. **(H)** Statistics of the relative gray value indicating the ATPIF1 protein expression in panel **(G)**. β-Actin served as a loading control. Ctrl means control. Three independent repeated experiments were performed. Student’s *t*-test was used to determine differences in panels **(B,D)**. One-way ANOVA with repeated measurement were used to determine differences in panels **(F,H)**. The data shown are the means ± SD, *n* = 3. * or ^#^ means *p* < 0.05, ** or ^##^ means *p* < 0.01. ns means no significant difference.

We sought to determine the downstream molecules that mediate sevoflurane-induced microglial inflammation and apoptosis, and we further found that sevoflurane treatment significantly upregulated ATPIF1 protein expression in mouse primary microglia (Figures 2A,B).

To confirm the function of ATPIF1, we overexpressed ATPIF1 in the microglial cell line BV2 and found upregulation of the inflammatory mediators COX-2 and TNF-α and activation of caspase-3 (Figures 2C,D). Conversely, knockdown of ATPIF1 expression in BV2 cells (Figures 2E,F) attenuated the inflammatory mediator upregulation and caspase-3 activation caused by sevoflurane treatment (Figures 2G,H). Therefore, we conclude that sevoflurane upregulates the expression of ATPIF1 to induce inflammation and caspase-3 activation.

### Adenosine Triphosphate Supplementation Ameliorates ATPase Inhibitory Factor 1- or Sevoflurane-Induced Inflammation and Caspase-3 Activation

**FIGURE 3:**
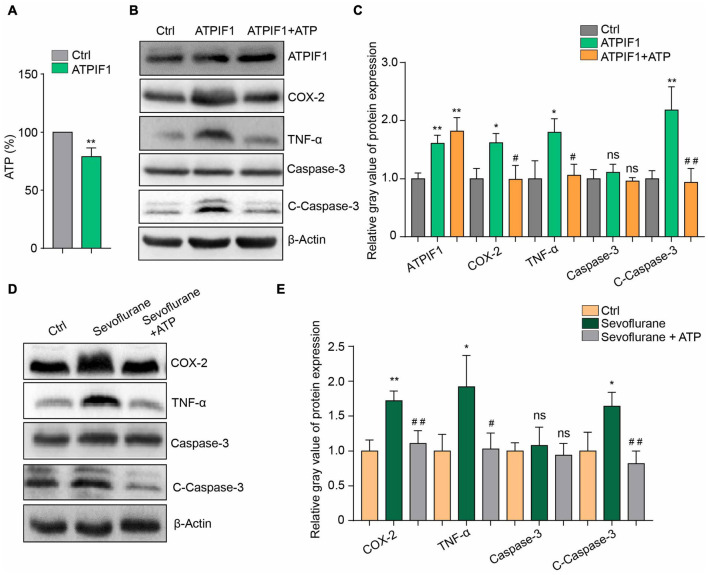
Additional ATP supplementation ameliorated ATPIF1- or sevoflurane-induced inflammation and caspase-3 activation in BV2 cells. **(A)** Overexpression of ATPIF1 decreased the intracellular ATP concentrations in BV2 cells. **(B)** ATP (50 μg/ml) ameliorated the ATPIF1 overexpression induced inflammation and caspase-3 activation in BV2 microglia. **(C)** Statistics of the relative gray value indicating the protein expression in panel **(B)**. **(D)** ATP also ameliorated sevoflurane treatment- induced inflammation and caspase-3 activation in BV2 microglia. **(E)** Statistics of the relative gray value indicating the protein expression in panel **(D)**. Ctrl means control. β-Actin served as a loading control. One-way ANOVA with repeated measurement were used to determine differences in panels **(C,E)**. The data shown are the means ± SD, *n* = 3, * or ^#^ means *p* < 0.05, ** or ^##^ means *p* < 0.01. ns means no significant difference.

To determine ATPIF1 function in microglia, we constructed BV2 microglial cells overexpressing ATPIF1 and found that overexpression of ATPIF1 decreased the intracellular ATP concentration (Figure 3A). This result not only indicated that ATP synthesis was inhibited but also suggested that the amount of ATP secreted outside the cell was reduced. Therefore, we added ATP to the culture medium to a final concentration of 50 μg/ml and found that ATP supplementation restored inflammatory upregulation and caspase-3 activation in BV2 microglia overexpressing ATPIF1 (Figures 3B,C) or treated with sevoflurane (Figures 3D,E). In summary, we found that extracellular supplementation with ATP ameliorates the inflammation and caspase-3 activation induced by ATPIF1 overexpression.

### Inflammation Regulates ATPase Inhibitory Factor 1 Expression

**FIGURE 4:**
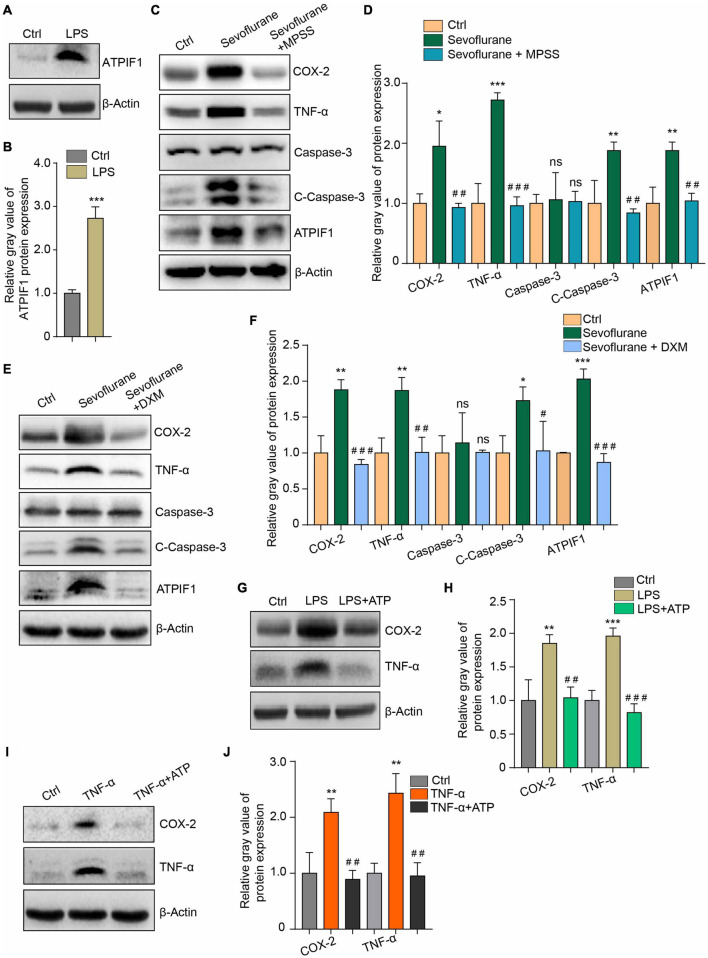
Inflammation regulates ATPIF1 expression and is attenuated by additional ATP supplementation in microglia. **(A)** LPS treatment upregulates ATPIF1 expression in microglia. **(B)** Statistics of the relative gray value indicating the ATPIF1 protein expression in panel **(A)**. **(C)** Methylprednisolone sodium succinate (MPSS) attenuated the COX-2 and TNF-α upregulation and promotion of caspase-3 activation caused by sevoflurane. **(D)** Statistics of the relative gray value indicating the protein expression in panel **(C)**. **(E)** Dexamethasone (DXM) also attenuated the COX-2 and TNF-α upregulation and promotion of caspase-3 activation caused by sevoflurane. **(F)** Statistics of the relative gray value indicating the protein expression in panel **(E)**. **(G)** ATP (50 μg/ml) ameliorated LPS induced inflammation in mouse primary microglia. **(H)** Statistics of the relative gray value indicating the protein expression in panel **(G)**. **(I)** ATP (50 μg/ml) ameliorated TNF-α-induced inflammation in mouse primary microglia. **(J)** Statistics of the relative gray value indicating the protein expression in panel **(I)**. Ctrl means control. β-Actin served as a loading control. Student’s *t*-test was used to determine differences in panel **(B)**. One-way ANOVA with repeated measurement were used to determine differences in panels **(D,F,H,J)**. The data shown are the means ± SD, *n* = 3. * or ^#^ means *p* < 0.05, ** or ^##^ means *p* < 0.01, *** or ^###^ means *p* < 0.001. ns means no significant difference.

Microglia can produce increased inflammatory mediators that can also further reactivate microglia through a positive feedback loop (Zhang et al., 2014; Bras et al., 2020). We found that 100 ng/mL lipopolysaccharide (LPS), which can activate microglia to induce inflammatory cytokine release, induced ATPIF1 upregulation in primary microglia (Figures 4A,B). This may indicate that a double positive feedback loop exists between the regulation of ATPIF1 expression and inflammation in microglia. Then, we used methylprednisolone sodium succinate (MPSS) (Figures 4C,D) and dexamethasone (DXM) (Figures 4E,F), which are anti-inflammatory drugs commonly used in clinical surgery, and found that they significantly inhibited the regulatory effects of sevoflurane on inflammation and caspase-3 activation in primary microglia. Additionally, 50 μg/ml ATP supplementation ameliorated LPS (Figures 4G,H)- or TNF-α (Figures 4I,J)-induced inflammation. Here, we confirmed the feedback loop between ATPIF1 expression and inflammation.

### Acute Adenosine Triphosphate Treatment Ameliorates the Anxious and Short-Term Memory Behaviors of the Postoperative Delirium Mouse Model

**FIGURE 5:**
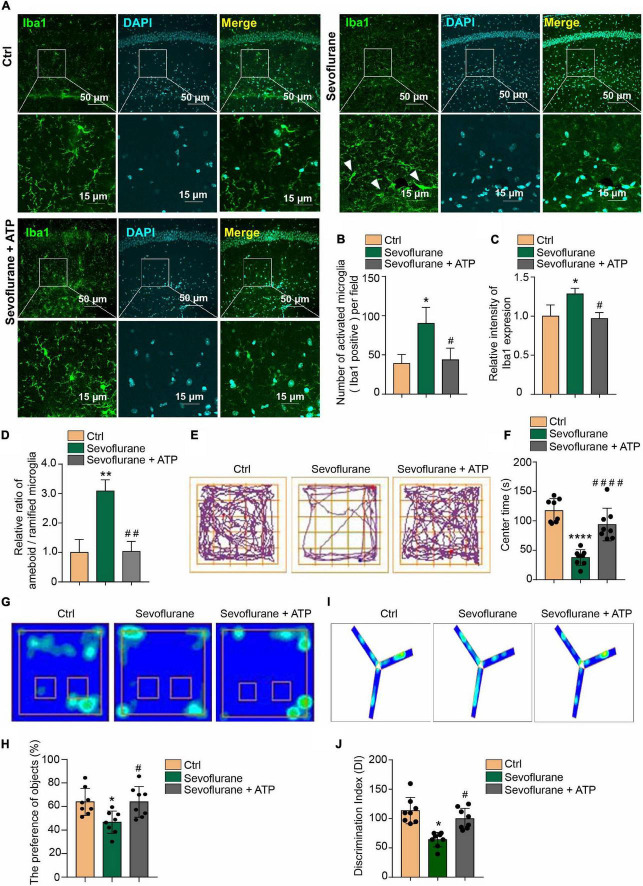
Attenuation of anxious and short-term memory behaviors by acute ATP treatment in POD mice. **(A)** Intraperitoneal (i.p.) injection of 2 mg/kg ATP ameliorated the 3% sevoflurane treatment inducing microglia activation as detected by Iba1 immunofluorescence staining in hippocampal tissue. The scale bar indicates 50 (upper row) and 15 μm (lower row), respectively. **(B)** ATP supplementary inhibited the increase of quantification of Iba1 + cell numbers and **(C)** intensity in sevoflurane-treated mice. **(D)** The ratio between amoeboid/ramified was also restored to be similar with the control groups by ATP supplementary in sevoflurane treatment mice. **(E,F)** Intraperitoneal (i.p.) injection of 2 mg/kg ATP ameliorated anxious behaviors in sevoflurane treatment POD mice, as detected by the open field test. **(G,H)** Novel object recognition and **(I,J)** Y-maze tests indicated that intraperitoneal (i.p.) injection of 2 mg/kg ATP ameliorated short-term memory impairment in the POD mouse model. One-way ANOVA with repeated measurement and *post hoc* analysis with Bonferroni were used to analyze the data presented in panels **(B–D,F,H,J)**. The data shown are the means ± SD [*n* = 3 in **(B–D)** *n* = 8 in **(E–J)**]. For all data, * and ^#^ indicate *p* < 0.05, ** and ^##^ indicate *p* < 0.01, and *** and ^###^ indicate *p* < 0.001.

Microglial inflammatory activation has been reported to induce cognitive impairment (Feng et al., 2017; Zhao et al., 2019). Cognitive impairment in POD is associated with anxiety, memory impairment and other symptoms (Gao X. et al., 2020; Pankaj et al., 2020; Ren et al., 2021). Therefore, according to our above findings in this study, we wanted to determine whether ATP supplementation can alleviate cognitive impairment to a certain extent. Therefore, we administered ATP (2 mg/kg) by intraperitoneal (i.p.) injection to POD mice. First, we found that only a single injection of ATP ameliorated the 3% sevoflurane treatment inducing microglia activation as detected by Iba1 immunofluorescence staining in hippocampus (Figure 5A). ATP supplementary inhibited the increase of quantification of Iba1 + cell numbers (Figure 5B). and intensity (Figure 5C) in sevoflurane-treated mice. The ratio between amoeboid/ramified was also restored to be similar with the control groups by ATP supplementary in sevoflurane treatment mice (Figure 5D). After confirming that ATP attenuated the microglia activation in POD mice, we performed the behavioral test and found that ATP supplementary ameliorated the anxious behaviors of the POD mice, as detected by the open field test (Figures 5E,F). Additionally, ATP ameliorated the short-term memory impairment in POD mice, which was detected by novel object recognition (Figures 5G,H) and Y-maze tests (Figures 5I,J). Based on the above results, we conclude that ATP supplementation significantly alleviates the two manifestations of mouse POD.

**FIGURE 6:**
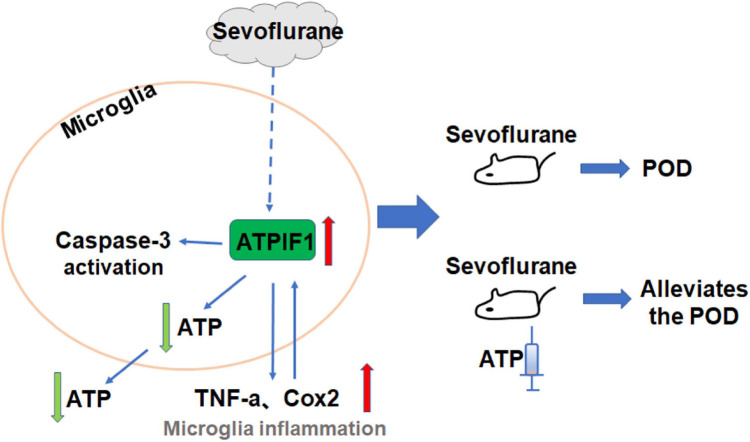
The hypothesized pathway indicating sevoflurane regulates microglial inflammatory responses and caspase-3 activation. Proposed mode pathway indicates that the sevoflurane promotes ATPIF1 expression in microglia to induce inflammatory factor upregulation and capsase-3 activation and decrease ATP level. Supplementation of ATP alleviated sevoflurane induced anxiety behavior and memory damage in POD mice.

Our results indicate that the clinical concentration of sevoflurane increases ATPIF1 expression in microglia to induce inflammatory factor upregulation and caspase-3 activation and decrease ATP level. Supplementation of ATP attenuates sevoflurane induced POD related anxiety behavior and memory damage in mice (Figure 6).

## Discussion

Postoperative delirium is a prevalent complication that is frequently observed after surgery and general anesthesia (Sieber et al., 2018; Kinjo et al., 2019). Generally, POD shows various symptoms, such as short-term memory impairment and anxiety (Smith et al., 2016; Jin et al., 2020). Although the activation of microglial inflammatory responses and apoptosis induced by anesthetics are thought to play a vital role in the occurrence of cognitive disorders, the mechanisms of these phenomena remain unclear. As indicated earlier, sevoflurane can induce microglial activation followed by inflammatory responses, and excessive microglial activation further leads to the loss of neurons; these are believed to be crucial factors in POD (Dai et al., 2015; Wang et al., 2021). Here, by targeting microglia, we confirmed that it could be activated by clinical concentrations of sevoflurane in the mouse brain. Additionally, *in vitro* treatment of primary microglia revealed elevated expression of inflammatory factors and activation of caspase-3. Previous studies have shown that some general anesthetics, such as sevoflurane and isoflurane, are highly relevant to the activation of microglia and apoptosis via the p38 MAPK pathway, NLRP3-caspase-1 pathway or NF-kB pathway (Yin et al., 2014; Wang et al., 2018; Yu et al., 2019). However, whether there is a particular molecule that can mediate both sevoflurane-induced microglial activation and apoptotic effects is still unclear. In the current study, we revealed for the first time that ATPIF1, a crucial protein that regulates ATP synthesis, may be involved in the pathogenesis of sevoflurane-induced microglial inflammation and apoptosis. In addition, our results indicate that exogenous supplementation with ATP may alleviate POD, which not only provides a better understanding of POD pathogenesis, but also suggests a novel alternative and relatively safe approach for attenuating sevoflurane-induced neurotoxicity.

Adenosine triphosphate is regarded not only as a biological energy source involved in many intracellular reactions but also as one of the signaling molecules in the CNS due to its actions on microglia (Färber and Kettenmann, 2006; Rivera et al., 2016). Under physiological conditions, maintenance of the stability of the extracellular ATP supply is essential for promotion of the normal functions of microglia. Some studies have reported that an increase in ATPIF1 levels is reflected in an elevated ATP content, whereas several other studies have suggested that upregulation of ATPIF1 promotes the inhibition of ATP production under some circumstances (Campanella et al., 2008; Kahancová et al., 2018, 2020). However, our findings demonstrated that overexpression of ATPIF1 resulted in a decreased ATP concentration; thus, we concluded that ATP secretion by the cells was correspondingly reduced. We clearly determined that exogenous supplementation with ATP attenuated the upregulation of proinflammatory cytokines in microglia and the activation of the caspase-3 triggered by excessive ATPIF1. In addition, our results indicated that sevoflurane can cause the downregulation of ATP content in microglia, which is consistent with previous studies showing that general anesthesia drugs can induce mitochondrial damage and neuronal apoptosis, resulting in reductions in intracellular ATP levels (Stadnicka and Bosnjak, 2003; Zhu et al., 2021). Furthermore, we found that exogenous supplementation with ATP can alleviate microglial inflammatory responses and apoptosis caused by sevoflurane anesthesia. In short, the extracellular ATP content is highly relevant to sevoflurane-induced inflammatory responses and proapoptotic effects in microglia.

Microglia can be activated by various stimuli, including LPS, TNF-α, IFN-γ, promoting the release of proinflammatory factors, and contributing to neuronal damage and degeneration (Glass et al., 2010). More specifically, when CNS homeostasis is persistently disrupted, the phagocytic activity of microglia is elevated, and this elevation is followed by excessive secretion of reactive oxygen and nitrogen species, proinflammatory chemokines and cytokines, often activating a paracrine or autocrine loop (Pocock and Liddle, 2001; Block and Hong, 2007). In our study, we observed that inflammatory stimuli further upregulated ATPIF1 expression, which revealed the existence of a positive feedback loop in the microglial inflammatory response. Furthermore, we found that intraoperative drugs such as DXM and MPSS, which are commonly used to ameliorate POD, were able to inhibit the inflammatory response of microglia and ameliorate the consequences caused by sevoflurane. Likewise, the inflammatory response due to LPS or TNF-α was alleviated by extracellular ATP supplementation. These results suggest that ATPIF1 acts as a potential mediator that participates in the positive feedback loop modulating the inflammatory response of microglia.

General anesthesia is a documented factor that directly results in neuronal apoptosis and is therefore considered to be a major cause of POD (Alam et al., 2018; Gao Y. et al., 2020). Through our studies on microglia, we also found that sevoflurane-induced apoptosis was significantly inhibited after the addition of ATP extracellularly. The above findings suggest that there may be a unified molecular mechanism involved in modulating the neurotoxicity of sevoflurane on microglia. Specifically, ATPIF1 can be considered an important mediator that contributes to the inflammatory response and apoptosis of microglia caused by sevoflurane. Previous studies have illustrated the neurotoxicity of general anesthetics on microglia and neurons. For instance, sevoflurane triggers the inflammatory response of microglia in the hippocampus by blocking the Wnt/β-Catenin/CaMKIV pathway (Wang et al., 2021). Sevoflurane also induced neuronal apoptosis through the PI3K/AKT/FOXO3a pathway (Dong et al., 2018). Our study reveals ATPIF1-mediated regulation of sevoflurane-based toxicity in microglia, which may provide mechanistic insights into pathologically diverse neurological disorders.

Clinically, POD has been confirmed to accelerate cognitive dysfunction and the onset of dementia (Vlisides and Avidan, 2019). Unfortunately, POD is not well understood, and there is limited evidence supporting the efficacy of intraoperative drugs such as DXM and MPSS, which are commonly used in the clinic to ameliorate POD (Vlisides and Avidan, 2019). As a result, whether there is a novel therapeutic strategy that can be used to alleviate POD-associated behavioral responses in order to protect the brain from excessive activation of microglia should be taken into consideration. In our study, we further demonstrated in animal experiments that the learning and memory impairments and the anxiety behaviors caused by sevoflurane treatment in mice can be significantly ameliorated by intraperitoneal injection of a relatively low concentration of ATP. In short, supplementation with ATP *in vivo* may attenuate POD-associated cognitive disorder and other behavioral changes.

In summary, the present study, which targeted the clinical issue of sevoflurane-induced POD, revealed that ATPIF1 acts as a negative regulator to induce the neurotoxicity of sevoflurane on microglia, further improving POD-associated behavioral responses. Additionally, we found that exogenous supplementation with ATP is beneficial for relieving pathological changes related to POD. These findings, which collectively indicate that ATPIF1 plays a novel role in the regulation of the inflammatory response and apoptosis of microglia caused by sevoflurane, suggest that therapeutic approaches designed to appropriately supplement ATP might be attractive POD prevention and treatment strategies.

## Data Availability Statement

The raw data supporting the conclusions of this article will be made available by the authors, without undue reservation.

## Ethics Statement

The animal protocol was approved by the Standing Committee on Animals at Shanghai Tenth People’s Hospital and mice were maintained in accordance with guidelines in the Laboratory Animal Center at Tongji University.

## Author Contributions

YX, GG, and XS carried out the experiment and analyzed the data. QL and CL jointly supervised the project and drafted the manuscript with input from all other authors. All authors contributed to the article and approved the submitted version.

## Conflict of Interest

The authors declare that the research was conducted in the absence of any commercial or financial relationships that could be construed as a potential conflict of interest.

## Publisher’s Note

All claims expressed in this article are solely those of the authors and do not necessarily represent those of their affiliated organizations, or those of the publisher, the editors and the reviewers. Any product that may be evaluated in this article, or claim that may be made by its manufacturer, is not guaranteed or endorsed by the publisher.
