# Corrigendum: ATPase Inhibitory Factor 1 Is Critical for Regulating Sevoflurane-Induced Microglial Inflammatory Responses and Caspase-3 Activation

**DOI:** 10.3389/fncel.2022.901582

**Published:** 2022-05-19

**Authors:** Yaru Xu, Ge Gao, Xiaoru Sun, Qidong Liu, Cheng Li

**Affiliations:** ^1^Department of Anesthesiology and Perioperative Medicine, Shanghai Fourth People's Hospital, School of Medicine, Tongji University, Shanghai, China; ^2^Translational Research Institute of Brain and Brain-Like Intelligence, Shanghai Fourth People's Hospital, School of Medicine, Tongji University, Shanghai, China; ^3^Clinical Research Center for Anesthesiology and Perioperative Medicine, Tongji University, Shanghai, China; ^4^Department of Anesthesiology, Shanghai Tenth People's Hospital, School of Medicine, Tongji University, Shanghai, China; ^5^Center for Translational Neurodegeneration and Regenerative Therapy, Shanghai Tenth People's Hospital, School of Medicine, Tongji University, Shanghai, China; ^6^Anesthesia and Brain Research Institute, Shanghai Tenth People's Hospital, School of Medicine, Tongji University, Shanghai, China; ^7^Key Laboratory of Spine and Spinal Cord Injury Repair and Regeneration of Ministry of Education, Orthopedic Department of Tongji Hospital, School of Medicine, Tongji University, Shanghai, China

**Keywords:** sevoflurane, postoperative delirium (POD), microglia, ATPIF1, ATP – adenosine triphosphate

In the original article, there was a mistake in the legend for Figure 5 as published. There were identification errors of notes **(E–J)** in the legend. And also in Figure 5, the Merge pictures of Ctrl and Sevoflurane + ATP groups are repeated in the original article. We corrected the Merge picture in the Ctrl group. The corrected Figure 5 appears below.

**Figure 5:**
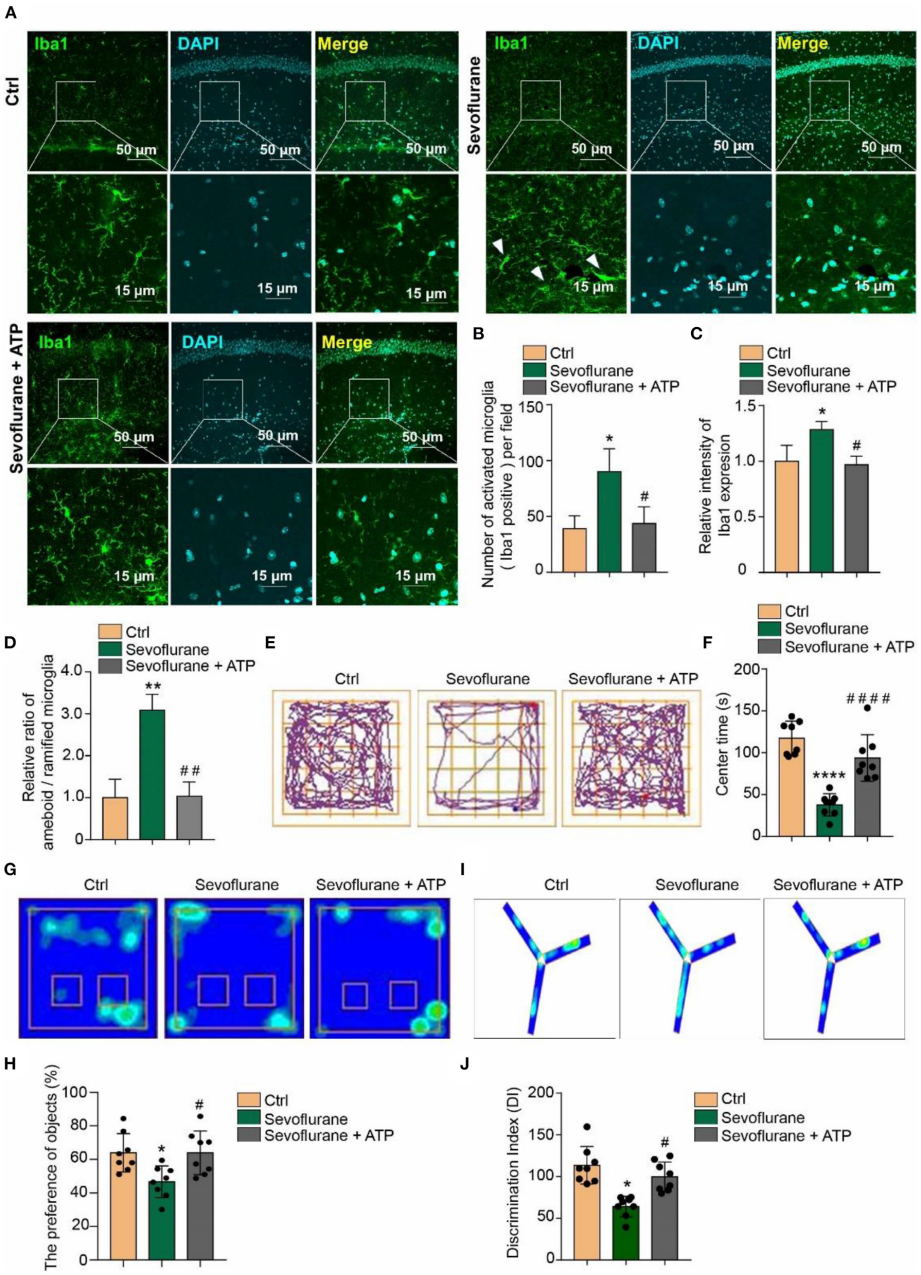
Attenuation of anxious and short-term memory behaviors by acute ATP treatment in POD mice. **(A)** Intraperitoneal (i.p.) injection of 2 mg/kg ATP ameliorated the 3% sevoflurane treatment inducing microglia activation as detected by Iba1 immunofluorescence staining in hippocampal tissue. The scale bar indicates 50 (upper row) and 15 μm (lower row), respectively. **(B)** ATP supplementary inhibited the increase of quantification of Iba1 + cell numbers and **(C)** intensity in sevoflurane-treated mice. **(D)** The ratio between amoeboid/ramified was also restored to be similar with the control groups by ATP supplementary in sevoflurane treatment mice. **(E,F)** Intraperitoneal (i.p.) injection of 2 mg/kg ATP ameliorated anxious behaviors in sevoflurane treatment POD mice, as detected by the open field test. **(G,H)** Novel object recognition and **(I,J)** Y-maze tests indicated that intraperitoneal (i.p.) injection of 2 mg/kg ATP ameliorated short-term memory impairment in the POD mouse model. One-way ANOVA with repeated measurement and *post hoc* analysis with Bonferroni were used to analyze the data presented in panels **(B–D,F,H,J)**. The data shown are the means ± SD [*n* = 3 in **(B–D)** *n* = 8 in **(E–J)**]. For all data, ^*^ and # indicate *p* < 0.05, ^**^ and ^##^ indicate *p* < 0.01, and ^***^ and ^###^ indicate *p* < 0.001.

The authors apologize for this error and state that this does not change the scientific conclusions of the article in any way. The original article has been updated.

## Publisher's Note

All claims expressed in this article are solely those of the authors and do not necessarily represent those of their affiliated organizations, or those of the publisher, the editors and the reviewers. Any product that may be evaluated in this article, or claim that may be made by its manufacturer, is not guaranteed or endorsed by the publisher.

